# High energy 1.53-cycle pulses via homogeneous post-compression in a single thin-plate

**DOI:** 10.1038/s41598-026-40980-y

**Published:** 2026-02-25

**Authors:** Gaudenis Jansonas, Dominykas Karvelis, Pija Gadonaitė, Arūnas Varanavičius

**Affiliations:** Laser Research Center, Vilnius University, Saulėtekio Ave. 10, LT-10223 Vilnius, Lithuania

**Keywords:** Optics and photonics, Physics

## Abstract

This study presents the investigation of the previously scarcely explored high energy few-cycle post-compression regime. Experimental shortening of the 5 mJ, 830 nm and 7.7 fs pulse with a flat-top spatial profile was achieved by nonlinear spectral broadening in a single 1 mm thick fused silica plate. The results show $\mathrm {3.8^{+0.20}_{-0.11}\,fs}$ pulse duration with a $\mathrm {64^{+2}_{-5}}\,\%$ relative peak power. The achieved spatial-spectral homogeneity was $97.1\,\%$. By employing the deformable mirror wavefront correction the Strehl ratio of 0.88 was shown even in the presence of a strong nonlinear interaction. It was demonstrated that a simple numerical model can predict output spectral properties well. The presented research might enable the future generation of high energy and quality single-cycle super-octave pulses.

## Introduction

In recent years, the pulse post-compression technique became a popular and useful tool for overcoming the bandwidth limitations of optical amplifiers^1^, thus enabling the growth of achievable peak power and intensity. In many cases, more importantly, the shortest obtainable high energy optical pulses are of immense value in the development of the secondary radiation sources of particles^2,3^, ultra violet (UV) light^4–6^ or efficient isolated attosecond pulses (IAP)^7–11^. The post-compression is based on the spectral broadening caused by the intensity-dependent refractive index called self-phase modulation (SPM)^1^.

Single^12^ and multiple thin plates^13^, gas-filled hollow core fibers (HCF)^14^, multi-pass cells (MPC)^15^ and the cascaded focus and compression (CASCADE)^16^ techniques were all successful in generating even the few-cycle pulses with often large compression factors (50 and more times) after the nonlinear spectral broadening and the residual chirp compensation.

For the post-compression technique, long initial pulse durations with high spectral broadening factors cause complicated output spectral shape and phase modulations. This creates temporal sidelobes even in transform-limited pulses and makes the envelope hardly compressible to the Fourier limit, thereby degrading the picosecond and femtosecond contrast^17^. Therefore, to produce the best quality (bell-like temporal shape) few-cycle wave-packets after the post-compression it is beneficial to start with the shortest possible pulses of the initial laser system. Currently, the broadest high-pulse-energy optical spectra (without the nonlinear broadening) are achieved by optical parametric chirped pulse amplification (OPCPA)^18^, where multi-TW and sub-10 fs pulses are usually obtained at around 800 nm central wavelength^19^. Here, we note that within the context of this work, the central wavelength is defined as the intensity-weighted mean wavelength. Furthermore, even sub-5 fs pulses were shown at the cost of additional complexity of two-color pumping^20,21^. This makes the OPCPA technology a very suitable candidate for exploring the few-cycle post-compression regime.

The nonlinear spectral broadening is an intensity-dependent process, meaning that for the Gaussian-like beam profiles the spectral shape and width should vary in space.

It turns out that this problem is solved in most of the current practical cases as it is only present in the high energy single-pass multi^22^ or single^12^ plate approaches and filamentation in gases^23^. In contrast, HCFs^24,25^, MPCs^26^ and the CASCADE^16^, when designed correctly, all show excellent spatial-spectral homogeneity. Furthermore, even spectral broadening in bulk can be homogeneous when operating below (or around) critical self-focusing peak powers and using long SPM media^27^ or employing near-field super-Gaussian beam profiles^28^. All of the presented SPM-based technologies are quite appealing for high peak power few-cycle pulse applications; however, most of these approaches have an inconvenient footprint scaling with increasing pulse energy and require niche tools.

This is mainly caused by the necessity of multiple SPM and re-compression stages, loose focusing or long propagation distances^13,16,29–33^. In comparison, the flat-top beam and a thin glass plate combination is a very straightforward approach, which currently allows performing the post-compression at record breaking joule energy levels^28,34–38^ while keeping the complexity and footprint at the same level as necessary for the operation of the employed laser system itself.

With these aforementioned energy records and simplicity in mind, we have

ventured into the pursuit of millijoule-level sub-5 fs pulses at around 800 nm central wavelength (sub-2 optical cycles)

by employing the single thin-plate technique. We start from an already short 7.7 fs pulse duration to achieve high temporal quality envelope after the anticipated twofold shortening.

In terms of high-order harmonic generation (HHG)^39,40^, the photon cut-off energy scales with roughly the inverse square root of the pulse duration of the driver^6^, providing a $\approx 40$ % extension in our case. Moreover, additional efficiency-increasing effect of nonadiabatic self-phase-matching^4^ can be used for $\approx 5$ fs duration pulses. During HHG, above a certain intensity threshold an attosecond pulse is generated every half-cycle of the electric field oscillation^41^. For IAP generation one needs to extract only a single attosecond pulse from the train, therefore, the efficiency of such process increases proportionally to the energy fraction carried by each half-cycle for a given gating technique, which, in our case, would nearly double the expected throughput. Furthermore, with sub-5 fs driver pulses an efficient over-saturation ionization gating technique^10^ can be used, which does not require additional optical elements when compared to a standard HHG setup. Finally, during the laser-plasma acceleration, the energy required to achieve the bubble regime^42,43^, which is needed for the production of high-quality relativistic electron beams, has a cubic scaling in terms of the initial pulse duration^2^. For this reason, a twofold pulse shortening would reduce the energy requirements by 8 times, allowing to bring down the foot-print of the laser system and operate at higher repetition rates. Therefore, further shortening the initial 7.7 fs pulse duration has a lot of value within the context of the secondary radiation sources.

## Methods

### Experimental setup

**Fig. 1:**
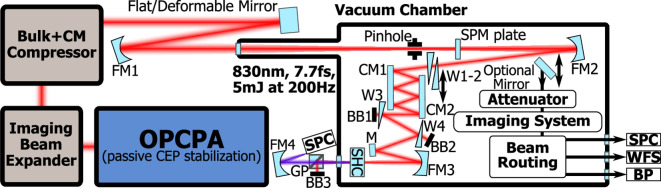
Principal optical layout. Here, OPCPA–optical parametric chirped pulse amplification, CEP–carrier-envelope phase, CM–chirped mirror(s), FM–focusing spherical mirror, SPM–self-phase modulation, W–wedge prism, BB–beam block, M–Mirror, SHC–second-harmonic crystal, GP–Glan-Thomson polarizer, SPC–spectrometer, WFS–wavefront sensor, BP–beam profiler.

The principal optical layout of the experiments is shown in Fig. 1.

Here, we have used a passively carrier-envelope phase-stabilized OPCPA system for our post-compression experiments. The previously reported core architecture^44^ was adopted for lower pump energy, divided into two picosecond amplification stages. The imaging spherical mirror beam expander, comprised of 300 mm and 2500 mm focal lengths, allowed us to ensure the relay imaging of the signal beam profile from the last amplification stage to the thin plate. By adjusting the distance between two curved mirrors we were able to tune the magnification of the image after the focus of a third focusing mirror (FM1).

The 1250 mm focal length spherical mirror (FM1) focused the approximately 30 mm diameter beam to the vacuum chamber with $(10^{-2}-10^{-3})$ mbar pressure. At the focal spot we have used a relatively large pinhole (around 1 mm in diameter) to remove high-frequency spatial modulations with insignificant losses in a similar way as reported before^28^.

After the spectral broadening in the 1 mm thick fused silica plate the beam was collimated and sent to the compressor. A pair of $10^{\circ }$ fused silica wedges (W1-2) were used for dispersion fine-tuning and the pulse measurement. Negative group delay dispersion was introduced with a commercial complementary chirped mirror pair (CM1 and CM2) for 800 nm (Few-Cycle Inc.), which induced on average -37.5 $\mathrm {fs^2}$ group delay dispersion and -23 $\mathrm {fs^3}$ third-order dispersion per single reflection.

### Pulse measurements

The initial pulse duration was measured to be 7.7 fs with a relative peak power (RPP, defined in the same way as the temporal Strehl ratio^45^) of 100 %. This means that approximately no fraction of the peak power was lost as a result of the uncompensated chirp with respect to the transform-limited pulse. The initial pulse measurement was performed inside the vacuum chamber with the second-harmonic generation (SHG) chirp scan^46^ (not shown in Fig. 1). A type-I 10 $\mathrm {\mu m}$ thick beta barium borate (BBO) crystal was used for the SHG process and the fundamental-harmonic was filtered out with an absorptive spectral filter. The chirp was varied with an acousto-optic programmable dispersive filter (AOPDF, Dazzler, Fastlite) before the high energy optical parametric amplifiers.

After the post-compression the beam was attenuated by using the first surface reflection of two wedge prisms (W3 and W4) and then subsequently focused with a spherical mirror (FM3). The focal spot was placed prior to the same (10 $\mathrm {\mu m}$ BBO) second-harmonic crystal (SHC) in order to avoid cross-phase modulation between the fundamental and second-harmonic waves in the fused silica substrate of the SHC. The second-harmonic radiation was almost fully separated from the fundamental wave by employing a calcite Glan-Thomson prism for UV spectral range (GP, Bernhard Halle Nachfl, GmbH). The beam was then refocused to a multi-mode fiber-coupled silicon (Si) detector-based spectrometer (Avantes) with an aluminum focusing mirror (FM4). Every presented spectrum was measured with intensity and wavelength calibrated spectrometers. The fundamental spectrum was measured in the same way by rotating the GP and attenuating the beam with reflective filters (Thorlabs). For the wavelength range beyond 1050 nm we have used an indium gallium arsenide (InGaAs) detector-based spectrometer (D-Vision, Sphere Ultrafast Photonics). The coupling of the whole beam to the spectrometer was ensured when measuring the spectra. For post-compressed pulse measurements we have varied the glass insertion length with W1-2 and registered the second-harmonic spectra generated in the SHC to perform the dispersion scan^47^ for the spectral phase retrieval only. For this task we have developed and employed a two-step retrieval algorithm. First, the differential evolution^48^ was used to get the prime approximation of the spectral phase, implemented in a very similar way as reported previously^49^. Next, the final refinement was performed by using the Nelder-Mead algorithm^50^ as implemented in the built-in Matlab function^51,52^.

The dispersion scan measurements showed rather good reproducibility, therefore, to estimate the measurement errors for pulse duration and RPP we have retrieved the spectral phase 25 times. We have removed strong outliers and calculated pulse duration and RPP for each measurement at the glass insertion value at which the mean retrieved spectral phase provides the highest relative peak power. From these values we have estimated the 95 % confidence bounds by calculating the standard deviation. The two standard deviation-long error interval was shifted from the mean so that the main measured value would represent the average measured spectral phase.

### Pre-compressor diagnostics

To characterize the output properties before the compressor we have manually inserted an optional pick-off mirror. The energy was again attenuated by reflecting the beam off two wedge prisms. A spherical mirror imaging system was employed to either image the SPM plate or the deformable mirror (ILAO STAR, Imagine Optic). For wavefront measurements we have employed a commercial Shack-Hartmann wavefront sensor (WFS, HASO, Imagine Optic). The deformable mirror correction was performed by running a commercial Strehl ratio optimization algorithm from Imagine Optic. The beam quality factor $\mathrm {M^2}$ of the output beam was quantified from the WFS intensity and phase measurement as described in the ISO 11146 standard, by simulating the beam (treated as a continuous wave) propagation after an idealized focusing element. For spectral measurements the whole beam was coupled into the fiber-connected spectrometers with a focusing mirror. In order to measure the spatial-spectral homogeneity we have translated the spectrometer-coupled fiber tip with 200 $\mathrm {\mu m}$ diameter core through the center of the beam in a horizontal (with respect to the optical table) direction. For the comparison of the spectral broadening measurements with the low intensity case we have chirped the pulses with AOPDF and this way stretched them around 140 times.

## Results

### Nonlinear spectral broadening in the few-cycle regime

**Fig. 2:**
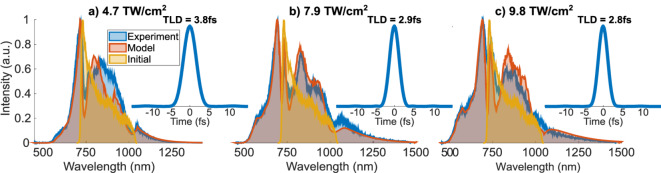
The illustration of the spectral broadening capabilities in the single 1 mm thick fused silica plate at different peak intensities (**a**–**c**). Experimental and numerical spectral broadening data are compared with the initial spectrum. The presented peak intensity values were only used as an input to the numerical model and were not determined experimentally. Here, TLD - transform-limited duration and a.u. - arbitrary units.

Figure 2 illustrates the main results of the spectral broadening, where output spectra were recorded at different peak intensities by simply translating the glass plate along the diverging beam propagation direction, where the uniform spatial profile was still maintained.

The low bandwidth broadening ratio (up to 2.75 times) and relatively thick (1 mm) nonlinear media with non-negligible dispersion resulted in quite smooth final spectra with low frequency modulation and gradual intensity decay to 0 from both sides. This is magnificently expressed in the provided transform-limited pulses, where the amplitude of the sidelobes only reach up to 0.7 % of the peak power. At the extremity, we have observed 2.8 fs pulse-supporting spectrum at the 760 nm central wavelength, which corresponds to 1.1 optical field oscillations throughout the full width at half maximum (FWHM) of the intensity envelope. However, we would argue that opting for $\ge$2.9 fs is a more practical choice, as for the broadest spectrum regime 19.4 % peak intensity increase only provides 3.5 % decrease in spectrally-limited pulse duration. Moreover, the requirement of permanent operation at $\approx$10 $\mathrm {\frac{TW}{cm^2}}$ peak intensity could decrease the overall lifetime of the thin plate and severely limit the average power of the system. On the other hand, a roughly twofold spectral broadening can be achieved at 4.7 $\mathrm {\frac{TW}{cm^2}}$, corresponding to the transform-limited duration (TLD) of $\mathrm {TLD=3.8}$ fs and 1.44 optical cycles at 794 nm carrier wavelength.

Furthermore, Fig. 2 offers the experimental data comparison with numerical simulations, which were obtained by using a simple one-dimensional nonlinear propagation model. Here, the input peak intensities and the linear chirp were varied to obtain the best visual fit. The numerical modeling was performed by solving the forward envelope equation^53^ with neglected transverse effects. The model included linear dispersion, frequency-dependent SPM, nonlinear refractive index dispersion^54,55^, delayed Raman response, multi-photon absorption and plasma generation. However, only the first two of the listed terms had a very significant impact under our experimental conditions. We have found that only a −7.5 $\mathrm {fs^2}$ initial chirp adjustment was necessary to replicate the features in the central region of the spectrum. This is not surprising, as the chirp was optimized with 10 $\mathrm {fs^2}$ steps for the broadest spectrum as observed by the Si detector-based spectrometer. The numerical simulations indicate that for this optimization criterion the pulse is required to be chirped. Despite the low dimensionality of our model, the numerical data rather closely agree with the experiment, even though the measurements were made in the few-cycle regime. The success of a one dimensional model was obviously expected as the input beam profile on target was close-to-uniform, nevertheless, the experimental confirmation provides substantiate validity.

### Pulse re-compression

In our experiments, the transmission of the compressor setup for the initial pulse was measured to be around 47 % with 89 % coupling efficiency to the vacuum chamber, meaning that the initial energy was reduced from 5 mJ to 2.09 mJ. We expect the full bandwidth losses to be slightly larger since the reflectivity of the chirped mirrors rather sharply decrease in value beyond 1100 nm, thus introducing additional 1.5–5 % of losses depending on the bandwidth of operation. Nevertheless, the low efficiency was primarily caused by uncoated glass optics and aluminum (Al) or silver (Ag) collimating and folding (not shown in Fig. 1) mirrors. This could be effortlessly avoided by replacing the off-the-shelf elements with custom-made optics or rearranging of the optical setup of the compressor. By avoiding the circumstantial metallic mirrors we could increase the efficiency to 73 % and by correctly coating all the glass optics the estimated throughput limit of 91 % could be reached. Here, while estimating the efficiency capabilities it was assumed that each of the 6 fused silica surfaces with correct dielectric coatings has 1 % losses and each of the 7 required dielectric mirror reflection has 0.5 % losses. Nevertheless, despite low experimental efficiency, 2.09 mJ sub-2 cycle pulses are suitable for a number of strong-field physics applications.

**Fig. 3:**
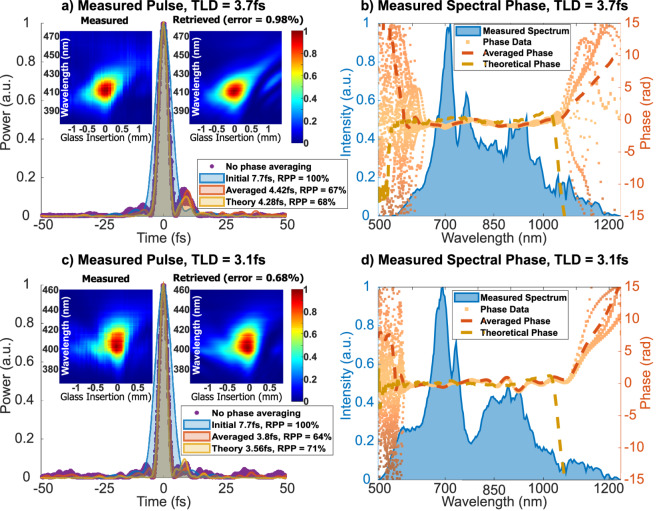
The illustration of the spectral phase measurement results for two different output bandwidths. (**a**, **c**)–pulse profiles with measured pulse duration and RPP values listed in the inset, (**b**, **d**)–spectral intensity and phase. Experimental results are shown in comparison to the theoretical predictions. The initial pulse is plotted for the reference. Corresponding measured and retrieved dispersion scan traces are shown in the inset of each temporal profile graph. Here, TLD - transform-limited duration, RPP - relative peak power and a.u. - arbitrary units.

The pulse compression results for achievable milestone pulse durations are shown in Fig. 3, where two pulses with different spectral bandwidths (supporting 3.7 fs and 3.1 fs) were compressed to the technological limit of our setup.

Spectral intensity and phase measurements, together with their respective theoretical phase curves, are shown in Fig. 3b and d. The theoretical curves were obtained by numerically modeling the nonlinear broadening and the subsequent linear dispersion of the fused silica wedges and complementary chirped mirror pairs. The corresponding temporal intensity profiles are presented in Fig. 3a and c. The primary pulse measurement data of the dispersion scan are presented in the insets of the pulse plots. The aforementioned data are shown in the form of measured and retrieved traces (corresponding to the mean measured spectral phase) of the frequency-doubled spectrum as a function of the relative glass thickness insertion. In both cases the measured and retrieved dispersion scan traces are very similar and exhibit low retrieval error. The finalized results show that the initial 7.7 fs and $\mathrm {RPP=100}$ % pulse was successfully shortened to $\mathrm {4.42^{+0.30}_{-0.05}}$ fs and $\mathrm {RPP=67^{+1}_{-6}}$% or even to $\mathrm {3.8^{+0.20}_{-0.11}}$ fs and $\mathrm {RPP=64^{+2}_{-5}}$ %. By calculating the temporal phase change of the electric field across the intensity FWHM, we determined that the measured pulse durations correspond to 1.70 and 1.53 optical cycles, respectively.

The results presented in Fig. 3 show that not every pulse and spectral phase feature was retrieved with full reliability. This can be perceived from the high uncertainty of the spectral phase at the ends of the power spectrum (Fig. 3b and d). The most likely cause of this phenomenon was the insufficient bandwidth of the second-harmonic generation crystal. The aforementioned data scattering indicates that we could not accurately use the spectral phase knowledge obtained from the frequency-doubled side-parts of the fundamental spectrum for our pulse measurements. Paradoxically, our results still provide crucial information that the specific wavelength portions of the pulse, which correspond to high spectral phase uncertainty, do not have a temporal overlap with the carrier-frequency region and, for this reason, are not compressed. Therefore, the true nature of the temporal side modulations of the pulse is unclear. Nonetheless, only the pulse width of the central peak is important for applications (when the amplitude of the side-pulses is roughly below 50 % of the peak power). To evaluate the trusted temporal envelope parts we rely on the performed theoretical analysis and error estimation. In Fig. 3a and c the experimentally determined pulse intensity profile data and the corresponding numerically calculated compressed pulses are illustrated. It is evident, that theoretical pulse durations and relative peak powers are rather close to the experimental ones, however, the agreement within error bars could not be shown. Inconsistencies aside, theoretical pulses represent the central peak and the first post-pulse very well, indicating that these features can be fully trusted. Additionally, the visual agreement with theory is better for temporally averaged pulses, rather than for the ones calculated from the mean measured spectral phase, nonetheless, the differences are minimal. On the other hand, the true form of the pre-pulses and low-amplitude post-pulses cannot be reliably determined. Regardless of this fact, the available data suggest that these features are of low amplitude and should not be of concern to the experimentalists. The theoretical ability to predict the main peak and the first post-pulse features of the measured pulses indicates that both measured pulse duration and RPP values are trustworthy, as the aforementioned temporal envelope characteristics obviously contribute the most to the emphasized quantification properties. Additionally, since the errors were estimated from the locally enormously ambiguous dataset, the presented duration and RPP values can be fully trusted within the presented uncertainty bounds.

The discussed results confirm the ability of the reported setup to reduce the pulse duration around 2 times, corresponding to 1.53 optical cycles. Regrettably, the output peak power was estimated to be reduced by about 35 %. Coincidentally, the peak power reduction is the same as the insufficient chirped mirror bandwidth-induced decrease in RPP. Nevertheless, the reduction in peak power was for the most part caused by circumstantial linear losses that could be avoided.

### Spatial properties

**Fig. 4:**
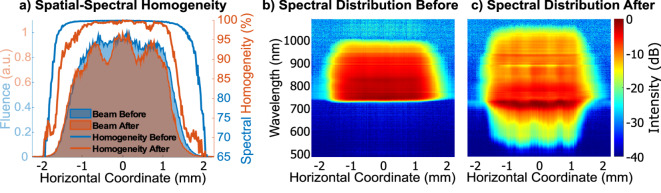
Spatial-spectral homogeneity properties before and after nonlinear spectral broadening. Here, a.u. - arbitrary units.

The demonstrated spectral broadening and pulse compression capture only part of the phenomenon, as free-space optical wave packets are inherently three-dimensional. Therefore, additional characterization is often required to prove the quality of the radiation. The quantification of the spatial-spectral couplings is presented in Fig. 4. The corresponding spectral broadening was close to the already presented (Fig. 3b and d) case.

Here (Fig. 4), we have presented the integral and local spatial-spectral properties before and after the nonlinear spectral broadening in the horizontal direction of the optical table, as observed from the image plane of the nonlinear glass plate. In Fig. 4a the statistical spectral homogeneity (defined in the scientific literature as the V parameter^56^) profile is shown. The initial fluence-weighted spectral homogeneity was 99.5 %. The almost perfect initial homogeneity degraded only to 97.1 % after the nonlinear process, which confirms that the spatial beam profile was adequately flat.

**Fig. 5:**
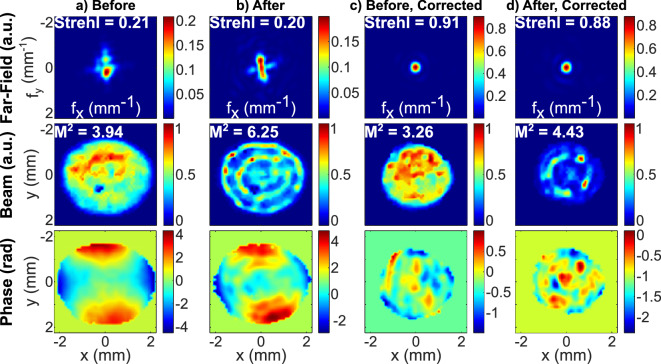
Integral spatial properties of the output beam from a Shack-Hartmann wavefront sensor measurements. Horizontal and vertical coordinates are denoted by x and y, while the horizontal and vertical spatial frequencies are denoted by $\mathrm {f_x}$ and $\mathrm {f_y}$, respectively. Strehl ratios and beam quality factors $\mathrm {M^2}$ are shown on far-field (calculated numerically) and near-field (beam) pictures. The near-field and far-field fluence profiles are displayed in arbitrary units (a.u.). Here, a) and b) show the spatial properties before and after nonlinear spectral broadening, as measured at the image plane of a thin-plate. c) and d) show the spatial properties before and after nonlinear spectral broadening, where the wavefront was corrected by the deformable mirror in each case. c) and d) cases were measured at the image plane of the deformable mirror.

The WFS was used for beam quality analysis. The measured beam properties before and after spectral broadening are illustrated in Fig. 5a and b.

Here, beam near-field fluence and wavefront profiles are presented as imaged from the plane of the thin plate of glass. From these measurements we have calculated the far-field fluence profiles, Strehl ratios and $\mathrm {M^2}$, which are presented in the same Figure. The Strehl ratio of an incident beam was around 0.21. As it can be seen from the phase profiles, the dominant aberration was determined to be the astigmatism. After the spectral broadening the irregularities of the initial beam profile got imprinted on to the wavefront, creating fine features in the spatial phase profile due to spatially varying SPM. Notably, after the spectral broadening the Strehl ratio was reduced by only about 5 %. In contrast, the calculated $\mathrm {M^2}$ degraded by nearly 60 %. We attribute this effect to the change in the recorded beam profile. The inner fluence minima and maxima could have appeared effectively more pronounced when measured by the Si detector matrix of the WFS. This could be caused by the wavelength-dependent responsivity to the varying levels of newly generated blue-side and red-side optical frequencies. This is supported by the fact that when accounting for the different wavelength contribution to the measured integral beam profile (Fig. 4a) the spatial intensity modulation has lower spatial frequency and depth. The outer high intensity ring could have appeared due to Kerr self-focusing, which could have been caused by steep intensity gradients at the edge of the beam.

The 60 % degradation of the beam quality factor could be tolerable as most systems of such caliber are primarily used for the far-field applications in order to achieve the highest possible intensity. Therefore, the ring structure, which usually appears in the far-field of the flat-top beam, gives rise to minimal inefficiencies to the secondary radiation generation. On the other hand, astigmatism and other wavefront aberrations cause a significant portion of the energy to be diffused around the peak of the far-field fluence profile which could have meaningful influence on the secondary radiation generation efficiency. For the compensation of the dominant astigmatism aberration we have used a deformable mirror. However, we must note that this could also be achieved by introducing a curved mirror system comprising of both concave and convex surfaces. The adaptive optic was placed right before the last focusing mirror prior to the vacuum chamber and its capabilities to correct the wavefront distortions in the nonlinear spectral broadening environment were tested afterwards. The wavefront correction results (before and after spectral broadening) are presented in Fig. 5c and d, only this time the WFS was placed at the image plane of the adaptive optic itself. The deformable mirror was able to remove the low-frequency wavefront distortions and improve the Strehl ratio by more than 4 times to 0.91. Gladly, the elevated focusability was preserved at high peak intensity and after the spectral broadening the Strehl ratio was measured to be 0.88 with most of the far-field energy concentrated at the main peak. Numerical calculations show that before the spectral broadening 83.3 % of the energy is concentrated within the main peak of the far-field beam profile. After the nonlinear process the energy concentration is reduced to 72.9 %. From the WFS measurements it was estimated that the peak fluence is reduced by 18.6 % after the post-compression, however, this is a reasonable trade-off for achieving the 1.53-cycle pulse duration regime.

## Discussion

By employing the SPM process within 1 mm thick fused silica plate with few-cycle initial wave-packets we have achieved smooth spectra that support high quality sub-3 fs pulses and span up to $\approx$1.5 octaves. Furthermore, the ability to tune the spectral broadening and pulse duration by translating the fused silica plate along the propagation direction was shown. The full compression of such-bandwidth-possessing pulses, especially in the presence of additional few millimeters of fused silica glass, is unfortunately, to the best of our knowledge, beyond the capabilities of the current state of the art chirped mirror technology. Nevertheless, the rising potential of generating more than 1.5 octaves spanning spectra may encourage further efforts to push the boundaries of chirped mirrors or even the development of entirely new spectral phase control approaches.

Our best efforts to compress the pulses with chirped mirror and glass wedge pairs resulted in $\mathrm {3.8^{+0.20}_{-0.11}}$ fs pulse duration with $\mathrm {RPP=64^{+2}_{-5}}$ % and linear losses-limited 47 % efficiency. Such a short pulse, when carrier-envelope phase is stable, was shown to be useful in the IAP generation^10,57^. In comparison with the scientific literature, similar pulse duration results (3.87 fs and superior RPP) were achieved with the same approach and a Gaussian beam profile^12^. Analogous 44 % energy efficiency was shown, however, most of the losses were accumulated due to the spatial filtering before the nonlinear process, while the spectral broadening and the compressor only amounted to about 11 % of wasted energy. This was achieved by allocating the glass wedges from the main beam to the pulse measurement device. Furthermore, the presented pulse duration was measured only from the center of the beam. In contrast, by placing the wedge compressor in the main optical path we have assured a fine dispersion tuning on the future experimental target for strong-field physics research and by imaging the flat-top beam profile to the thin nonlinear plate allowed us to measure the average 1.53-cycle pulse of the whole beam.

The simple one dimensional nonlinear propagation model was shown to replicate the measured 1.5 octaves spanning spectra. The same model could predict the measured pulse shape well, however, since numerical simulations suggest that the dominant factor in the compressor was uncompensated linear dispersion, it is difficult to make claims that a simple numerical apparatus is enough to fully design such few-cycle high energy post-compression systems. Nevertheless, currently there is no indication, that our approach could lead to errors of dispersion management implementation. Repeating the measurements and replicating them with theory when using a compressor which would provide at least $\textrm{RPP}=90$ % could fully confirm the effectiveness of our simple model.

The use of a quite uniform spatial profile led to a very tiny average spatial-spectral homogeneity degradation after the spectral broadening from 99.5 to 97.1 % in the most significant wavelength range below 1050 nm, suggesting, that high spatial-temporal homogeneity is also possible. Finally, the ability to correct for wavefront distortions even after the spectral broadening was confirmed. The use of the deformable mirror in the near-field thin-plate post-compression approach was already reported, however, the final Strehl ratio was described to be 0.52^38^. In contrast, we have measured the value of 0.88 in our case, which indicates a significant improvement and supports the fact that linear wavefront corrections could work even in the presence of a strong nonlinear process.

To the best of our knowledge, the single thin-plate post-compression approach was shown to successfully provide 1.53-cycle pulses in the 5 mJ uniform spatial profile regime for the first time. The focusability and spatial-spectral homogeneity were well preserved after a strong nonlinear process. The output spectral properties could be well replicated with a simple one-dimensional nonlinear propagation model. The employed free space architecture provides a convenient footprint scaling, which implies the possibility to adopt this technique and make use of the presented results when developing joule energy level few-cycle systems.

## Data Availability

The datasets generated and/or analyzed during this study are available from the corresponding author upon reasonable request.
